# Supplementary material for: The asymmetric effects of climate risk on higher-moment connectedness among carbon, energy and metals markets
Source: Nat Commun. 2023 Nov 7;14:7157. doi: 10.1038/s41467-023-42925-9 (PMC10630388; doi:10.1038/s41467-023-42925-9)
Supplement: Supplementary file 1 — Supplementary Information [file 41467_2023_42925_MOESM1_ESM.pdf]

Supplementary Materials for

The asymmetric effects of climate risk on higher-moment connectedness among carbon, energy and metals markets

Yuqin Zhou[1], **Shan Wu[2*]**, Zhenhua Liu[3], Lavinia Rognone[4,5]

[1]*School of Economics and Management, Chongqing Normal University, Chongqing, China.*

[2]*School of Finance, Nanjing University of Finance and Economics, Nanjing, China.*

[3]*School of Economics and Management, China University of Mining and Technology, Xuzhou, China.*

[4]*University of Edinburgh Business School, The University of Edinburgh, Edinburgh EH8 9JS, UK*

[5]*Alliance Manchester Business School, The University of Manchester, Manchester M15 6PB, UK*

[*] Corresponding author. Email: wushaniam@outlook.com.

Supplementary note 1: The previous research about the impact of climate risk on carbon, energy, and metals markets.

Supplementary note 2: The previous research about spillovers among the carbon, energy, and metals markets

Supplementary Table 1: The relevant literature on the spillover effects of the carbon, energy and metals markets, including their applied methods, markets, time periods, and key findings.

Supplementary Table 2: The parameter estimation for the GJRSK model. ***, **, and * denote 1%, 5%, and 10% level of significance, respectively.

Supplementary Table 3: The static skewness spillover connectedness based on the DY method and BK model (%). "TCI"presents the total spillover index. The directional spillover received of each market is denoted by "FROM", and transmitted is denoted by "TO" or "TO_ABS". The *jk* th value is the directional connectedness from *k* to *j*.

Supplementary Table 4: The static kurtosis spillover connectedness based on the DY method and BK model (%). "TCI" presents the total spillover index. The directional spillover received of each market is denoted by "FROM", and transmitted is denoted by "TO" or "TO_ABS". The *jk* th value is the directional connectedness from *k* to *j*.

Supplementary Table 5: Summary statistics of returns.The table shows the sample minimums (Min.), medians, means, maximums (Max.), skewness, kurtosis, standard deviations (Std. Dev), Jarque-Bera (J-B) tests which is the normality test of Jarque and Bera[1], Augmented Dickey-Fuller(ADF) tests which is the unit root test of Dickey and Fuller[2] and ARCH-LM test for the ARCH effect for up to the 10th order of the twelve returns series of carbon, energy and metals markets. *** and ** denote 1% and 5% levels of significance, respectively.

Supplementary Figure 1: The time-varying conditional return, variance, skewness and kurtosis of each market.The top panels represent the return, variance, skewness and kurtosis sequence of different markets, such as "EUA return" represents the time-varying conditional return of EUA.

Supplementary Figure 2: The dynamic net skewness time-frequency spillover effects. The plots are drawn by the monthly mean of dynamic net directional spillovers. The black horizontal line represents $y=0$. "Net directional spillovers" is the aggregate net directional spillovers of the DY model. "Short-term" and "Long-term" is the net directional spillover in the short and long-term horizons of BK model, respectively. The top panels represent each market.

Supplementary Figure 3: The dynamic net kurtosis spillover effect in the time-frequency domain. The plots are drawn by the monthly mean of dynamic net directional spillovers. The black horizontal line represents $y=0$. "Net directional spillovers" is the aggregate net directional spillovers of the DY model. "Short-term" and "Long-term" is the net directional spillover in the short and long-term horizons of BK model, respectively. The top panels represent each market.

Supplementary Figure 4: Net-pairwise directional connectedness before the COVID-19 pandemic period. These figures show the 66 pairs of carbon, energy and metals markets. The nodes represent each market, and the thickness of the edge shows the degree of the net-pairwise directional connectedness. The arrows going from markets *i* to *j* represent net spillovers, that is, the contribution of market *i* to market *j* is greater than that of market *j* to market *i*. "Net-pairwise directional spillovers" is the aggregate net-pairwise directional spillovers of the DY model. The "short-term" and "long-term" is the net-pairwise directional spillover in the short and long-term horizons of BK model, respectively.The top panels represent the volatility, skewness and kurtosis network in different frequencies, such as "Volatility (short-term)" represents the network drawn by

the net-pairwise directional volatility connectedness in the short-term horizons of BK model before the COVID-19 pandemic period.

Supplementary Figure 5: Net-pairwise directional connectedness during the COVID-19 pandemic period. These figures show the 66 pairs of carbon, energy and metals markets. The nodes represent each market, and the thickness of the edge shows the degree of the net-pairwise directional connectedness. The arrows going from markets *i* to *j* represent net spillovers, that is, the contribution of market *i* to market *j* is greater than that of market *j* to market *i*. “Net-pairwise directional spillovers” is the aggregate net-pairwise directional spillovers of the DY model. The “short-term” and “long-term” is the net-pairwise directional spillover in the short and long-term horizons of BK model, respectively. The top panels represent the volatility, skewness and kurtosis network in different frequencies, such as “Volatility (short-term)” represents the network drawn by the net-pairwise directional volatility connectedness in the short-term horizons of BK model during the COVID-19 pandemic period.

Supplementary Figure 6: Overall volatility spillover index using different rolling-window sizes. In order to avoid extreme values from masking other period trends, we drew the plots with the monthly mean of dynamic total connectedness. The top panel “Overall spillovers” is the dynamic total spillover index of the DY model. “Short-term” and “Long-term” is the dynamic frequency connectedness on the band:3.14 to 0.14 and 0.14 to 0 of BK model, respectively.

Supplementary Figure 7: Coefficient Comparisons for the quantile-on-quantile (QQ) and quantile regression (QR) method of the impact of physical risk index(PRI) on total spillover index(TCI). “Overall spillovers” is the dynamic total spillover index of the DY model. The “short-term” and “long-term” is the dynamic frequency connectedness on the band:3.14 to 0.14 and 0.14 to 0 of BK model, respectively. Top panels show the volatility, skewness, and kurtosis spillover effects in different frequencies, such as “Volatility (short-term)” represents the coefficients got by the impact of PRI on TCI in the short-term horizon based on QQ and QR model.

Supplementary Figure 8: Coefficient Comparisons for the quantile-on-quantile (QQ) and quantile regression (QR) method of the impact of transfer risk index(TRI) on total spillover index(TCI). “Overall spillovers” is the dynamic total spillover index of the DY model. The “short-term” and “long-term” is the dynamic frequency connectedness on the band:3.14 to 0.14 and 0.14 to 0 of BK model, respectively. The top panels show the volatility, skewness, and kurtosis spillover effects in different frequencies, such as “Volatility (short-term)” represents the coefficients obtained by the impact of TRI on TCI in the short-term horizon based on QQ and QR model.

Supplementary Figure 9: Heat map of pairwise correlations of each market’s returns. The numbers represent the pairwise correlation between pairs of assets, and the darker color represents a stronger correlation.

Supplementary note 1: The previous research about the impact of climate risk on carbon, energy, and metals markets.

In recent years, many scholars have begun to address the impact of climate risk on the economic and financial sectors[3-4]. Some scholars focus on how climate change affects the carbon, energy, and metals markets. On the one hand, existing studies explore the predictive role or the dynamic impact of climate change on the price and fluctuations of carbon, energy and metals markets. Climate uncertainty is an important driver of carbon emission price fluctuations[5-6], and including climate change in the carbon price prediction framework can significantly improve the accuracy of carbon market price prediction[7]. At the same time, climate uncertainty has a non-linear impact on crude oil and natural gas prices[8], and can also improve the prediction accuracy of renewable energy index volatility[9], putting some inflationary pressure on commodities such as metals[10]. For example, Gupta and Pierdzioch[11] test the impact of climate risk factors on the realized volatility forecasts of gold and other metal price, showing that climate risk factors improve the accuracy of out-of-sample forecast prices. Furthermore, researchers have categorically classified climate risks into physical risk and transition risk. A study conducted by Karmakar et al.[12] has demonstrated the significant predictive capability of physical risks for gold volume, particularly at 5- and 22-day-ahead horizons. Moreover, Salisu et al.[13] have uncovered noteworthy associations between the return volatility of gold and climate risks. Specifically, they found a positive and significant relationship between return volatility and transition risk, while a negative and significant relationship was observed with physical risk.

On the other hand, some scholars have studied the impact of climate change on the supply and demand of carbon, energy and metals markets. For example, Babiker[14] explored the impact of the relocation of production and energy-intensive enterprises on carbon emissions under climate policy change, and it showed that greenhouse gas control policies in industrialized countries would actually lead to higher global carbon emissions; Watari et al.[15] showed that global inventory of metals currently in use need to converge from about 4 tons to about 7 tons per capital to contribute to the industrial sector's emissions reduction targets; Tang et al.[16] investigated the future demand for lithium, cobalt and nickel in the Dutch electric vehicle market in different scenarios due to climate policy implementation, and the results showed that the demand for lithium and nickel will continue to grow by 2040, while the cobalt demand trend will start to decline after 2030; Shang et al.[17] showed that climate policy uncertainty reduces the demand for non-renewable energy and Lam et al.[18] showed a linear relationship between Singapore's temperature and domestic energy consumption, and a non-linear relationship in Hong Kong. In addition, Khalfaoui et al.[19] studied the spillover and connectivity network of the green index, carbon price, general and climate uncertainty. A study conducted by Mao et al.[20] using network analysis suggests that commodity markets, such as energy and metals, appear to be more sensitive to climate-related information.

Supplementary note 2: The previous research about spillovers among the carbon, energy, and metals markets

As carbon emissions trading has become one of the mainstream ways to solve climate change, more and more scholars have begun to study the correlation between the carbon trading market and the stock market[21-23], energy market[24-28], bitcoin market[29], and other financial markets[30-31]. Among them, Supplementary Table 1 shows the relevant literature on the spillover effects of the carbon, energy and metals markets, including their applied methods, markets, time periods, and key findings. It is evident from Supplementary Table 1 that scholars have shown considerable interest in exploring the relationship between carbon, energy, and metal markets, and the findings suggest significant spillover effects among these markets. From the perspective of method, the scholars mainly used the spillover index model built by Diebold and Yilmaz[32-33](DY model) and its related extension model, namely the frequency connectedness method proposed by Barunik and Krehlik[34] (BK model), which can quantify the risk spillover effect from the perspective of time domain and frequency domain simultaneously. Ando et al.[35] constructed the Quantile VAR model to better explore the tail risk spillover effects. As the heterogeneity of participants in the carbon, energy and metals markets, there are differences in their investment duration, risk appetite, constraints and investment objectives. For example, some speculators and hedge funds are more focused on the short-term performance of the market, while large institutional investors are more concerned about the long-term operation of the market[36]. Therefore, this paper decomposes the time-domain risk spillover effects of the carbon, energy and metals markets into different frequency-domain.

However, the existing literature has paid more attention to the return and volatility spillover effect among carbon, energy and metals markets. The return spillover is used to depict the price linkage between markets and measure market integration, and the volatility spillover is used to measure the risk contagion effect between markets. For example, Jiang and Chen[37] used the DY and BK model to study the return time-frequency spillover effects between metal, energy and carbon markets; Chen et al.[38] used the Quantile VAR model to study the volatility tail risk connectedness. However, financial market returns are characterized by peak and thick tail, and high order (skewness and kurtosis) risk is an important part of financial risk management[39]. The skewness spillover effects refer to the increase in the probability of a sharp decline or rise in other markets caused by the sharp fall or rise in the price of one market, and the kurtosis spillover effects reflect the increased possibility of causing similar extreme risk events in other markets after extreme risk events occur in one market. While, the study of high order risk spillover effect has not received similar attention. Cui and Maghyereh[40] examine the interconnectedness of higher-order moment risks between oil and commodity futures, while Zhou et al.[41] investigate the spillover effects of higher-moment tail risks among carbon, energy, and non-ferrous metals markets. In contrast to their studies, our research specifically focuses on the time-frequency spillover effects of higher-order moments. Although Bouri et al.[42] investigated the connectedness of volatility, skewness, and kurtosis between precious metals and energy markets in both the time and frequency domains, our study delves into the time-frequency spillover effects of higher-order moments across carbon, energy, and metals markets. Additionally, we consider the impact of the leverage effect when calculating volatility, skewness, and kurtosis. Then, we use the GJRSK model and BK model to discuss the time-frequency volatility, skewness, and kurtosis risk connectedness among carbon, energy and metals markets.

Supplementary Table 1: The relevant literature on the spillover effects of the carbon, energy and metals markets, including their applied methods, markets, time periods, and key findings.

Authors	Methodology	Main Markets	Period	Main Findings
Adekoya et al.[29]	DY and BK model	Carbon, gas, oil, copper, gold, silver, and stocks markets	2009.10-2020.10	The connectedness between the carbon market and others is non-trivial and heterogeneous.
Chen et al.[38]	Quantile VAR model	Carbon, gas, oil, gold, silver, copper, aluminum, lead, zinc, and nickel markets	2008.4.1-2021.10.29	The connectedness between energy, metal, and carbon markets is time-varying.
Jang and Chen[43]	DY and BK model	Carbon, coal, oil, new energy, iron, aluminum, cement, and plastic markets	2015.1.1-2021.7.31	Total connectedness is larger in the short term and enhanced after the COVID-19 outbreak.
Jiang and Chen[37]	DY and BK model	Carbon, coal, gas, oil, gold, silver, copper, and aluminum markets	2014.1.1-2022.3.31	The carbon market is increasingly linked to metal and energy markets over time.
Zhou et al.[41]	Quantile VAR model	Carbon, coal, gas, oil, copper, aluminum, lead, zinc, nickel, and Tin markets	2015.7.1-2022.2.28	There are significant risk spillover effects among carbon, energy and nonferrous metal markets.
Qi et al.[44]	BK model	Carbon, energy, oil & fats, non-ferrous metals, petrochemical, and softs markets	2013.9.2-2022.9.30	Carbon market are more influenced by other commodity markets.

Supplementary Table 2: The parameter estimation for the GJRSK model. ***, **, and * denote 1%, 5%, and 10% level of significance, respectively.

Parameter		EUA	Oil	Gas	Coal	Gold	Silver	Copper	Aluminum	Zinc	Nickel	Tin	Lead
Mean equation	α_1	-0.04**	0.10***	0.10***	-0.09***	0.00	-0.02	-0.02	-0.03	-0.06***	-0.04**	-0.04**	0.01
Variance equation	β_0	0.40***	0.36***	1.44***	0.04**	0.00	0.04***	0.01	0.23***	0.02	0.21***	0.04**	0.25***
	β_1	0.10***	0.01	0.02	0.02	0.03	0.07***	0.01	0.02	0.01	0.05**	0.16***	0.08***
	β_2	0.07***	0.13***	0.18***	0.00	0.00	0.00	0.01	0.17***	0.01	0.00	0.00	0.00
	β_3	0.82***	0.86***	0.80***	0.97***	0.96***	0.91***	0.98***	0.73 ***	0.98***	0.89***	0.84***	0.80***
Skewness equation	γ_0	0.02	-0.07***	0.02	-0.01	-0.08***	0.01	0.00	0.03*	0.01	-0.01	-0.08***	-0.01
	γ_1	-0.01	-0.11***	0.00	0.00	0.00	-0.11***	0.01	0.01	-0.01	0.00	0.00	0.01
	γ_2	0.01	0.11***	0.00	0.00	0.00	0.12***	0.02	0.05**	0.00	0.00	0.07***	-0.04 **
	γ_3	0.61***	-0.03*	0.81***	0.72***	-0.46***	0.29***	-0.02	0.68***	-0.59***	0.34***	0.01	0.46***
Kurtosis equation	δ_0	1.10***	3.34***	0.55***	3.94***	0.55***	1.93***	3.42***	1.17***	2.07***	3.45***	3.53***	2.22***
	δ_1	0.00	0.00	0.00	0.00	0.00	0.00	0.00	0.01	0.00	0.00	0.00	0.02
	δ_2	0.00	0.01	0.04**	0.01	0.00	0.01	0.01	0.04**	0.03	0.01	0.03	0.00
	δ_3	0.69***	0.01	0.84***	0.12***	0.84***	0.44***	0.00	0.62***	0.37***	0.00	0.01	0.31***

Supplementary Table 3: The static skewness spillover connectedness based on the DY method and BK model (%). "TCI" presents the total spillover index. The directional spillover received of each market is denoted by "FROM", and transmitted is denoted by "TO" or "TO_ABS". The *jk* th value is the directional connectedness from *k* to *j*.

Model		EUA	Oil	Gas	Coal	Gold	Silver	Copper	Aluminum	Zinc	Nickel	Tin	Lead	FROM
Panel A：DY(2012)	EUA	98.34	0.01	0.14	0.34	0.41	0.11	0.12	0.16	0.02	0.11	0.05	0.19	0.14
	Oil	0.10	99.26	0.09	0.30	0.03	0.06	0.03	0.05	0.00	0.04	0.02	0.03	0.06
	Gas	0.91	0.24	97.54	0.18	0.02	0.04	0.04	0.89	0.03	0.02	0.06	0.03	0.21
	Coal	1.03	0.06	0.16	98.00	0.01	0.03	0.05	0.60	0.01	0.02	0.02	0.01	0.17
	Gold	0.54	0.03	0.01	2.05	95.70	0.15	0.41	0.16	0.37	0.09	0.20	0.28	0.36
	Silver	0.20	0.06	0.01	1.88	0.15	96.56	0.05	0.49	0.04	0.37	0.03	0.15	0.29
	Copper	1.82	0.01	0.11	0.05	0.15	0.27	70.33	0.88	1.93	3.83	0.38	20.23	2.47
	Aluminum	0.66	0.04	0.03	0.74	0.04	0.08	1.41	94.02	0.94	1.05	0.40	0.59	0.50
	Zinc	0.17	0.30	0.02	0.09	0.77	0.05	1.70	8.27	71.14	0.84	12.60	4.05	2.40
	Nickel	0.07	0.01	0.62	0.02	0.05	0.15	3.70	2.31	1.13	90.80	0.27	0.86	0.77
	Tin	0.16	0.23	0.09	0.52	0.24	0.27	0.33	15.65	11.52	0.33	64.78	5.87	2.93
	Lead	0.65	0.04	0.04	0.19	0.20	0.58	2.44	4.41	4.84	0.94	7.18	78.50	1.79
	TO	0.53	0.09	0.11	0.53	0.17	0.15	0.86	2.82	1.74	0.64	1.77	2.69	TCI=12.08
Panel B：BK(2018) Frequency 1 (High frequency): 1day to 22 days	EUA	78.47	0.01	0.11	0.29	0.33	0.11	0.12	0.13	0.01	0.09	0.04	0.15	0.12
	Oil	0.08	94.51	0.05	0.21	0.02	0.06	0.03	0.03	0.00	0.04	0.02	0.03	0.05
	Gas	0.34	0.14	58.11	0.08	0.01	0.02	0.02	0.36	0.02	0.01	0.05	0.02	0.09
	Coal	0.57	0.04	0.10	68.43	0.00	0.02	0.02	0.27	0.00	0.01	0.01	0.01	0.09
	Gold	0.53	0.03	0.01	2.03	93.85	0.15	0.39	0.16	0.36	0.08	0.20	0.28	0.35
	Silver	0.16	0.06	0.01	1.73	0.14	87.11	0.04	0.38	0.04	0.29	0.03	0.14	0.25
	Copper	1.46	0.01	0.09	0.05	0.14	0.26	66.39	0.82	1.82	3.42	0.34	18.72	2.26
	Aluminum	0.32	0.04	0.02	0.48	0.03	0.07	1.02	74.08	0.79	0.85	0.29	0.42	0.36
	Zinc	0.17	0.30	0.02	0.08	0.77	0.05	1.66	8.08	70.29	0.83	12.43	3.97	2.36

	Nickel	0.07	0.01	0.31	0.01	0.05	0.12	3.28	1.47	1.06	80.40	0.26	0.73	0.61
	Tin	0.12	0.23	0.09	0.36	0.24	0.26	0.31	15.01	11.06	0.33	61.45	5.55	2.80
	Lead	0.53	0.04	0.04	0.14	0.17	0.47	2.05	3.91	4.31	0.77	6.21	68.84	1.55
	TO_ABS	0.36	0.07	0.07	0.45	0.16	0.13	0.75	2.55	1.62	0.56	1.66	2.50	TCI=10.89
Panel B：BK(2018) Frequency 1 (Low frequency): 22 days to infinity	EUA	19.87	0.00	0.03	0.05	0.08	0.00	0.00	0.02	0.01	0.03	0.01	0.03	0.02
	Oil	0.02	4.75	0.04	0.08	0.00	0.00	0.00	0.02	0.00	0.00	0.00	0.00	0.01
	Gas	0.57	0.10	39.42	0.10	0.01	0.02	0.02	0.53	0.01	0.01	0.01	0.01	0.12
	Coal	0.46	0.02	0.06	29.57	0.00	0.01	0.03	0.33	0.00	0.01	0.00	0.01	0.08
	Gold	0.01	0.00	0.00	0.02	1.85	0.00	0.02	0.00	0.01	0.01	0.00	0.00	0.01
	Silver	0.04	0.00	0.00	0.15	0.00	9.44	0.01	0.11	0.00	0.08	0.00	0.02	0.03
	Copper	0.36	0.00	0.01	0.00	0.01	0.01	3.94	0.06	0.11	0.42	0.04	1.51	0.21
	Aluminum	0.34	0.00	0.01	0.26	0.01	0.01	0.39	19.94	0.15	0.20	0.11	0.17	0.14
	Zinc	0.00	0.00	0.00	0.02	0.00	0.00	0.04	0.19	0.85	0.02	0.17	0.08	0.04
	Nickel	0.01	0.00	0.31	0.01	0.00	0.03	0.42	0.84	0.07	10.40	0.00	0.13	0.15
	Tin	0.04	0.00	0.00	0.16	0.00	0.01	0.02	0.64	0.46	0.00	3.33	0.32	0.14
	Lead	0.12	0.00	0.00	0.05	0.02	0.11	0.39	0.50	0.53	0.17	0.98	9.66	0.24
	TO_ABS	0.16	0.01	0.04	0.08	0.01	0.02	0.11	0.27	0.11	0.08	0.11	0.19	TCI=1.19

Supplementary Table 4: The static kurtosis spillover connectedness based on the DY method and BK model (%). “TCI” presents the total spillover index. The directional spillover received of each market is denoted by “FROM”, and transmitted is denoted by “TO” or “TO_ABS”. The *jk* th value is the directional connectedness from *k* to *j*.

Model		EUA	Oil	Gas	Coal	Gold	Silver	Copper	Aluminum	Zinc	Nickel	Tin	Lead	FROM
	EUA	94.60	0.03	3.71	0.01	0.03	0.85	0.51	0.04	0.03	0.01	0.05	0.14	0.45
	Oil	0.03	68.80	0.09	0.01	0.09	0.04	0.30	10.06	11.91	0.07	7.58	1.00	2.60
	Gas	3.27	0.04	95.28	0.01	0.18	0.08	0.01	0.49	0.31	0.17	0.09	0.07	0.39
	Coal	0.01	0.02	0.04	99.78	0.00	0.01	0.00	0.04	0.05	0.02	0.02	0.01	0.02
	Gold	0.02	0.01	0.06	0.00	82.12	17.22	0.12	0.02	0.18	0.11	0.11	0.03	1.49
	Silver	0.34	0.01	0.06	0.00	18.95	79.61	0.06	0.07	0.36	0.21	0.07	0.23	1.70
Panel A：DY(2012)	Copper	0.78	0.33	0.03	0.00	0.75	6.73	75.74	0.08	0.15	1.26	0.06	14.08	2.02
	Aluminum	0.15	0.23	2.37	0.02	0.28	0.08	0.22	95.89	0.24	0.10	0.18	0.24	0.34
	Zinc	0.05	6.91	0.69	0.01	0.16	0.23	0.07	27.20	40.03	0.09	19.81	4.75	5.00
	Nickel	0.14	0.15	0.03	0.01	0.02	0.17	0.70	0.40	0.45	97.66	0.18	0.09	0.19
	Tin	0.04	5.39	0.30	0.00	0.10	0.03	0.02	19.36	24.25	0.01	48.72	1.79	4.27
	Lead	0.72	0.79	0.01	0.01	0.19	3.61	0.10	2.87	7.42	0.07	2.60	81.62	1.53
	TO	0.46	1.16	0.62	0.01	1.73	2.42	0.18	5.05	3.78	0.18	2.56	1.87	TCI=20.01
	EUA	70.25	0.02	2.28	0.01	0.02	0.55	0.39	0.02	0.01	0.01	0.04	0.12	0.29
	Oil	0.03	65.02	0.05	0.01	0.06	0.03	0.28	9.29	11.40	0.07	7.25	0.92	2.45
	Gas	1.56	0.03	47.27	0.01	0.03	0.02	0.01	0.16	0.14	0.07	0.05	0.03	0.18
Panel B：BK(2018)<br>Frequency 1<br>(High frequency):<br>1day to 22 days	Coal	0.01	0.02	0.02	93.62	0.00	0.00	0.00	0.03	0.04	0.01	0.02	0.01	0.01
	Gold	0.01	0.01	0.01	0.00	41.62	8.82	0.06	0.02	0.10	0.04	0.06	0.01	0.76
	Silver	0.30	0.01	0.02	0.00	16.03	68.93	0.05	0.07	0.32	0.17	0.06	0.22	1.44
	Copper	0.70	0.32	0.01	0.00	0.66	5.91	71.82	0.07	0.13	1.10	0.05	13.40	1.86
	Aluminum	0.06	0.22	0.88	0.02	0.07	0.07	0.14	73.07	0.22	0.09	0.12	0.21	0.18
	Zinc	0.03	6.31	0.26	0.01	0.14	0.20	0.05	23.23	36.44	0.08	18.10	4.13	4.38

	Nickel	0.11	0.15	0.02	0.01	0.02	0.17	0.66	0.28	0.44	91.58	0.16	0.09	0.17
	Tin	0.03	5.17	0.13	0.00	0.09	0.02	0.02	18.06	23.27	0.01	46.53	1.66	4.04
	Lead	0.67	0.74	0.01	0.01	0.12	2.95	0.08	2.46	6.82	0.06	2.39	74.25	1.36
	TO_ABS	0.29	1.08	0.31	0.01	1.44	1.56	0.15	4.47	3.57	0.14	2.36	1.73	TCI=17.12
Panel B：Bk(2018) Frequency 1 (Low frequency): 22 days to infinity	EUA	24.35	0.00	1.43	0.00	0.01	0.30	0.12	0.02	0.01	0.00	0.01	0.02	0.16
	Oil	0.00	3.79	0.05	0.00	0.03	0.01	0.02	0.77	0.51	0.00	0.32	0.08	0.15
	Gas	1.71	0.01	48.01	0.00	0.15	0.06	0.01	0.32	0.17	0.10	0.03	0.04	0.22
	Coal	0.00	0.00	0.02	6.16	0.00	0.00	0.00	0.00	0.01	0.00	0.00	0.00	0.00
	Gold	0.01	0.00	0.05	0.00	40.50	8.40	0.07	0.01	0.08	0.06	0.05	0.01	0.73
	Silver	0.04	0.00	0.04	0.00	2.92	10.69	0.01	0.00	0.05	0.05	0.02	0.01	0.26
	Copper	0.08	0.01	0.02	0.00	0.10	0.82	3.92	0.01	0.01	0.16	0.00	0.68	0.16
	Aluminum	0.09	0.01	1.49	0.01	0.21	0.01	0.07	22.81	0.02	0.01	0.05	0.03	0.17
	Zinc	0.02	0.60	0.43	0.00	0.02	0.03	0.02	3.97	3.58	0.01	1.71	0.62	0.62
	Nickel	0.03	0.00	0.01	0.00	0.00	0.00	0.04	0.13	0.01	6.08	0.02	0.00	0.02
	Tin	0.01	0.22	0.16	0.00	0.01	0.00	0.00	1.30	0.98	0.00	2.18	0.13	0.23
	Lead	0.05	0.05	0.00	0.00	0.06	0.66	0.01	0.41	0.61	0.01	0.22	7.37	0.17
	TO_ABS	0.17	0.08	0.31	0.00	0.29	0.86	0.03	0.58	0.20	0.03	0.20	0.14	TCI=2.89

Supplementary Table 5: The sample minimums (Min.), medians, means, maximums (Max.), skewness, kurtosis, standard deviations (Std. Dev), Jarque-Bera (J-B) tests which is the normality test of Jarque and Bera[1], Augmented Dickey-Fuller(ADF) tests which is the unit root test of Dickey and Fuller[2] and ARCH-LM test for the ARCH effect for up to the 10th order of the twelve returns series of carbon, energy and metals markets. *** and ** denote 1% and 5% levels of significance, respectively.

	Min.	Median	Mean	Max.	Skewness	Kurtosis	Std. Dev	J-B	ADF	LM(10)
EUA	-17.69	0.13	0.19	20.43	-0.07	4.99	3.08	1722.25***	-12.29***	112.42***
Oil	-24.59	0.19	0.10	37.66	1.37	26.38	3.21	48545.20***	-9.80***	461.01***
Gas	-26.80	0.00	0.24	46.03	1.75	16.40	5.08	19407.13***	-12.26***	259.13***
Coal	-41.54	0.00	0.12	38.57	0.92	79.75	2.74	439089.02***	-12.09***	255.13***
Gold	-5.68	0.05	0.03	6.49	-0.01	4.34	0.94	1303.44***	-11.85***	123.52***
Silver	-15.02	0.02	0.02	8.27	-0.47	7.86	1.72	4323.01***	-11.24***	156.71***
Copper	-7.99	0.06	0.03	5.37	-0.21	2.03	1.27	297.11***	-11.13***	84.72***
Aluminum	-7.05	0.00	0.03	5.49	-0.17	2.48	1.21	434.44***	-12.47***	241.52***
Zinc	-9.66	0.04	0.03	10.28	0.07	2.72	1.58	511.53***	-11.79***	40.24***
Nickel	-11.99	0.06	0.03	14.99	-0.04	4.64	1.97	1491.15***	-12.19***	299.71***
Tin	-17.60	0.07	0.04	9.02	-1.51	17.87	1.47	22670.88***	-11.93***	160.52***
Lead	-7.56	0.02	0.01	8.39	0.04	2.08	1.40	299.73***	-12.35***	17.21**

Supplementary Figure 1: The time-varying conditional return, variance, skewness and kurtosis of each market. The top panels represents the return, variance, skewness and kurtosis sequence of different markets, such as "EUA return" represents the time-varying conditional return of EUA.

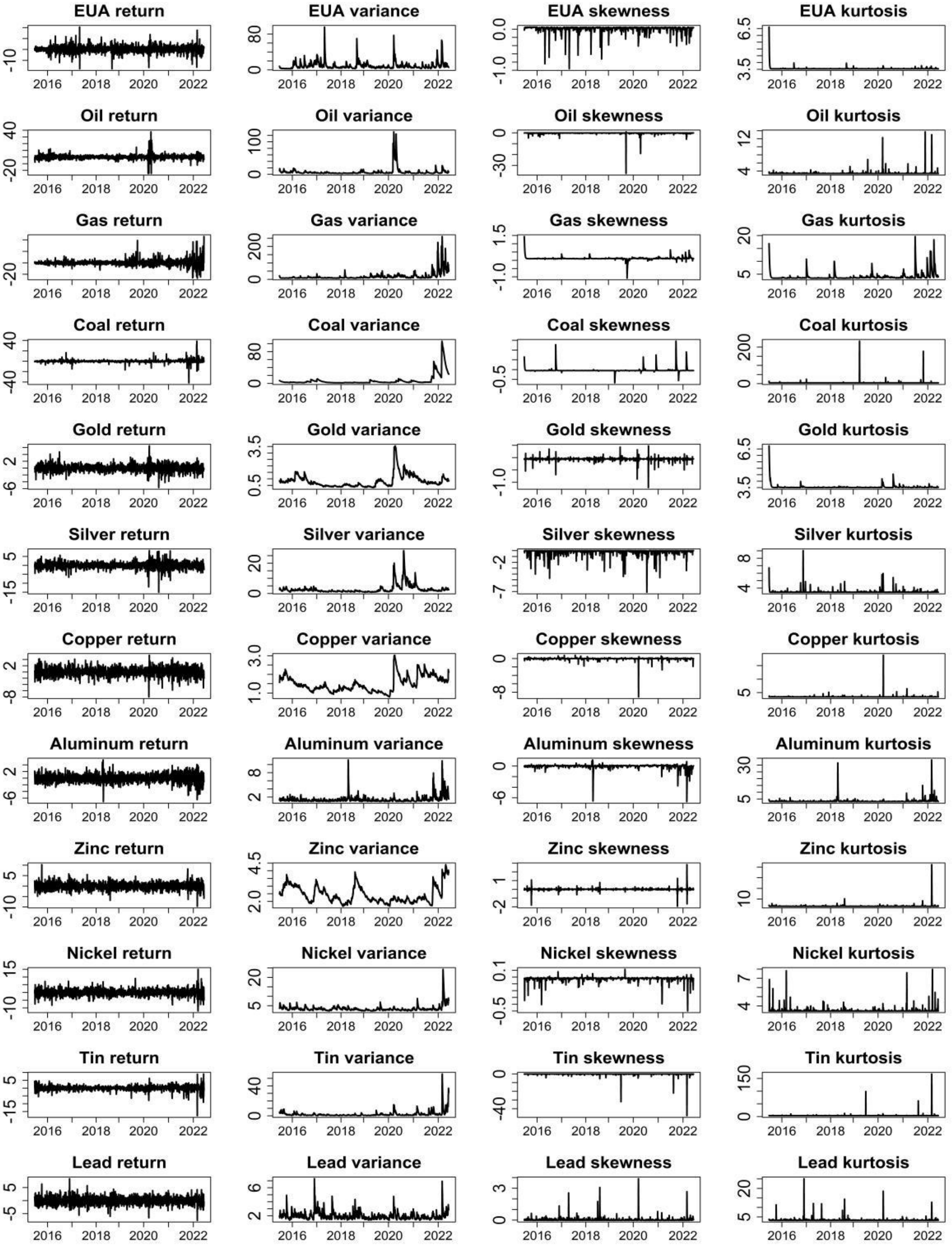

Supplementary Figure 2: The dynamic net skewness time-frequency spillover effects. The plots

are drawn by the monthly mean of dynamic net directional spillovers. The black horizontal line represents *y=0*. "Net directional spillovers" is the aggregate net directional spillovers of the DY model. "Short-term" and "Long-term" is the net directional spillover in the short and long-term horizons of BK model, respectively. The top panels represent each market.

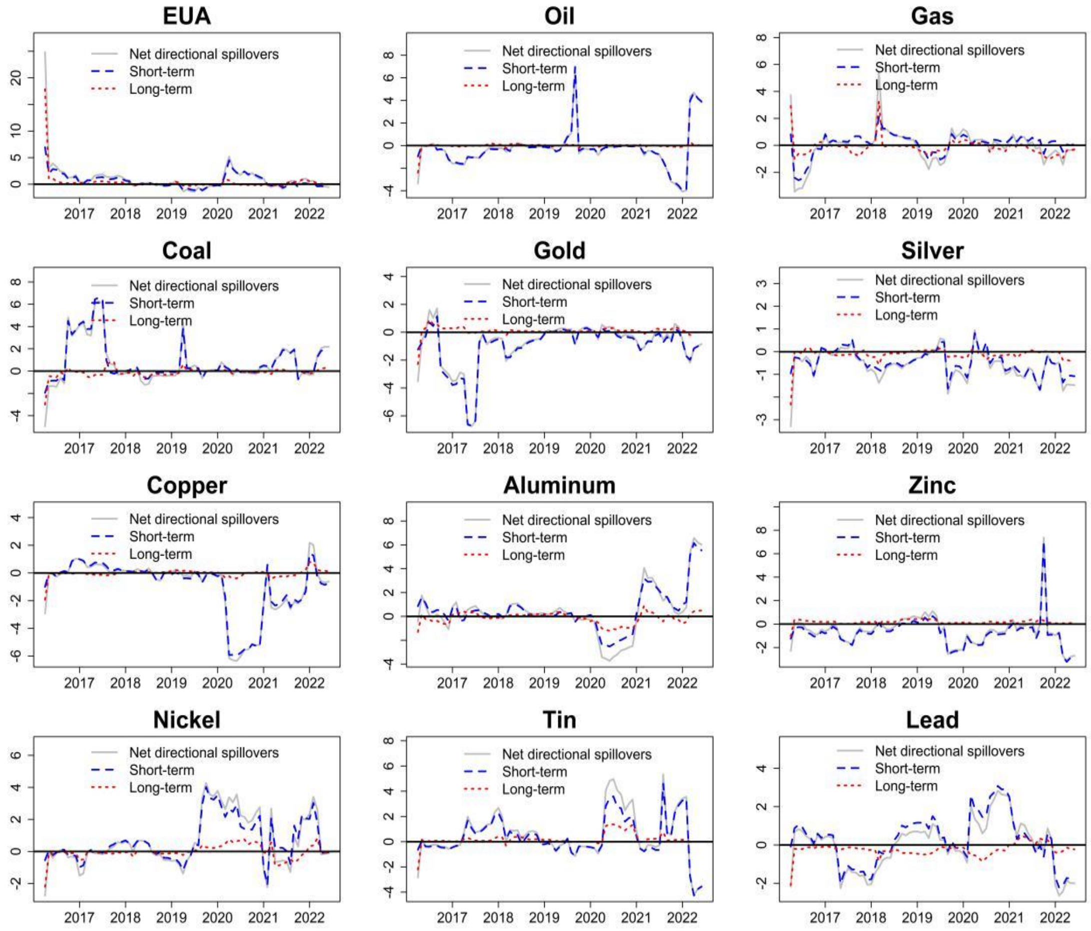

Supplementary Figure 3: The dynamic net kurtosis spillover effect in the time-frequency domain.

The plots are drawn by the monthly mean of dynamic net directional spillovers. The black horizontal line represents *y=0*. “Net directional spillovers” is the aggregate net directional spillovers of the DY model. “Short-term” and “Long-term” is the net directional spillover in the short and long-term horizons of BK model, respectively. The top panels represent each market.

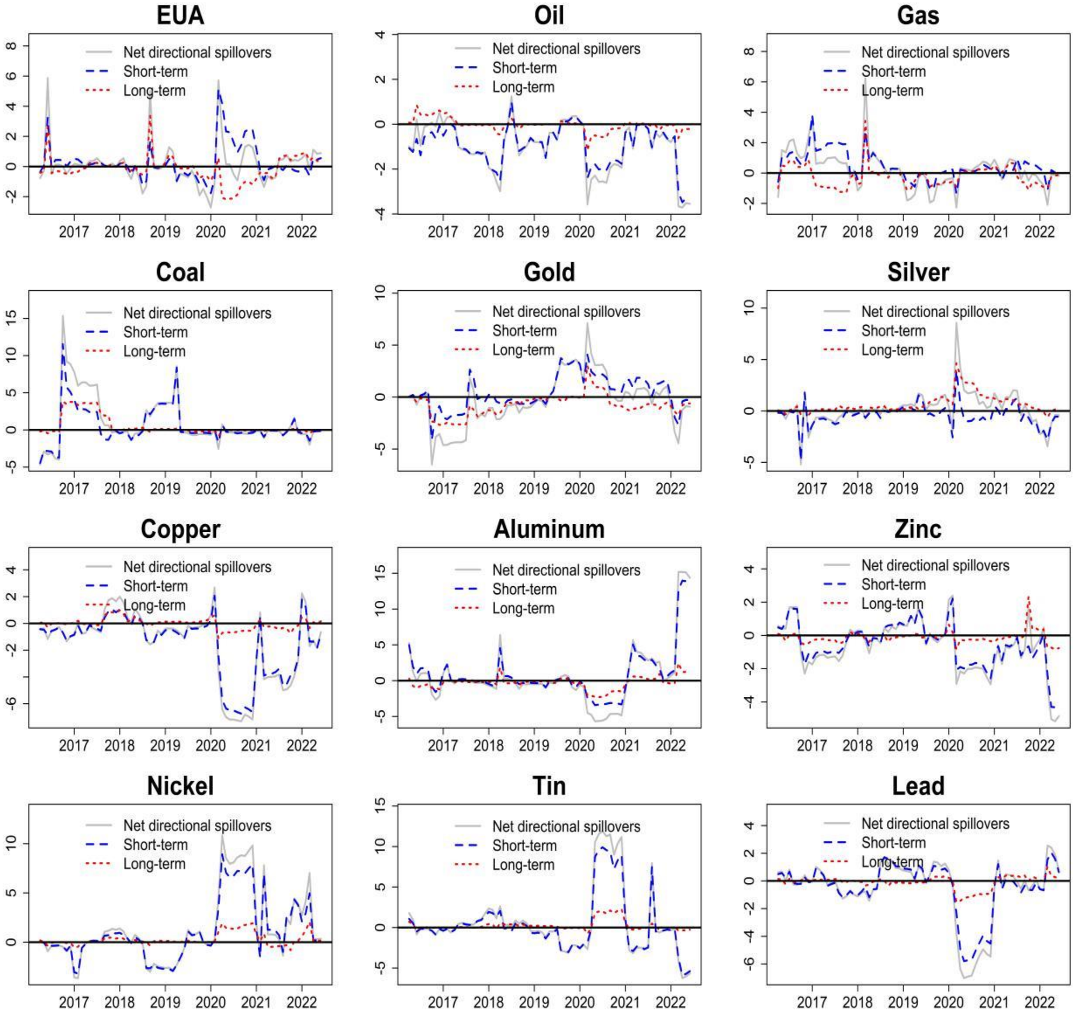

Supplementary Figure 4: Net-pairwise directional connectedness before the COVID-19

pandemic period. These figures show the 66 pairs of carbon, energy and metals markets. The nodes represent each market, and the thickness of the edge shows the degree of the net-pairwise directional connectedness. The arrows going from markets *i* to *j* represent net spillovers, that is, the contribution of market *i* to market *j* is greater than that of market *j* to market *i*. “Net-pairwise directional spillovers” is the aggregate net-pairwise directional spillovers of the DY model. The “short-term” and “long-term” is the net-pairwise directional spillover in the short and long-term horizons of BK model, respectively. The top panels represent the volatility, skewness and kurtosis network in different frequencies, such as “Volatility (short-term)” represents the network drawn by the net-pairwise directional volatility connectedness in the short-term horizons of BK model before the COVID-19 pandemic period.

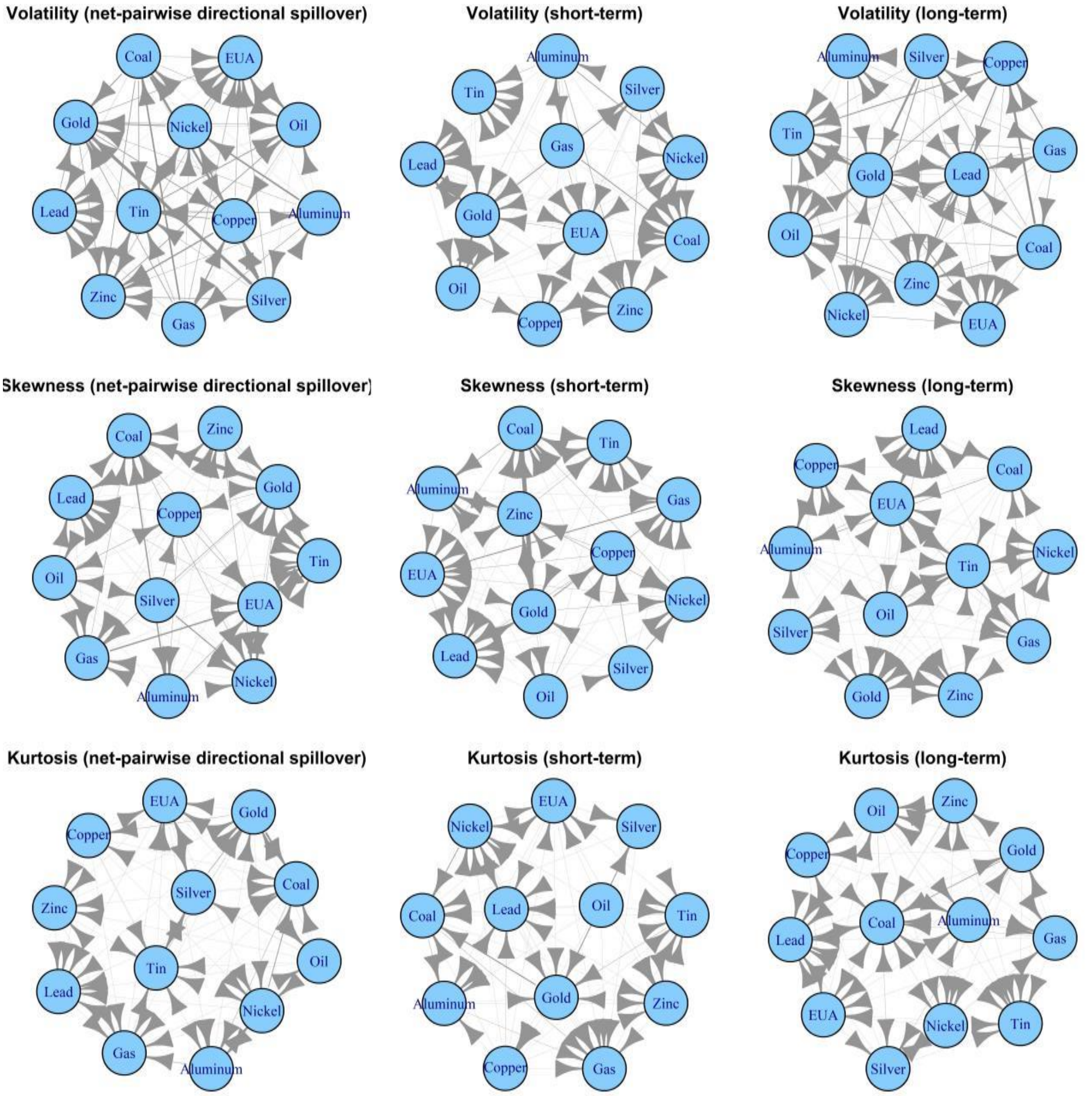

Supplementary Figure 5: Net-pairwise directional connectedness during the COVID-19

pandemic period. These figures show the 66 pairs of carbon, energy and metals markets. The nodes represent each market, and the thickness of the edge shows the degree of the net-pairwise directional connectedness. The arrows going from markets *i* to *j* represent net spillovers, that is, the contribution of market *i* to market *j* is greater than that of market *j* to market *i*. "Net-pairwise directional spillovers" is the aggregate net-pairwise directional spillovers of the DY model. The "short-term" and "long-term" is the net-pairwise directional spillover in the short and long-term horizons of BK model, respectively. The top panels represent the volatility, skewness and kurtosis network in different frequencies, such as "Volatility (short-term)" represents the network drawn by the net-pairwise directional volatility connectedness in the short-term horizons of BK model during the COVID-19 pandemic period.

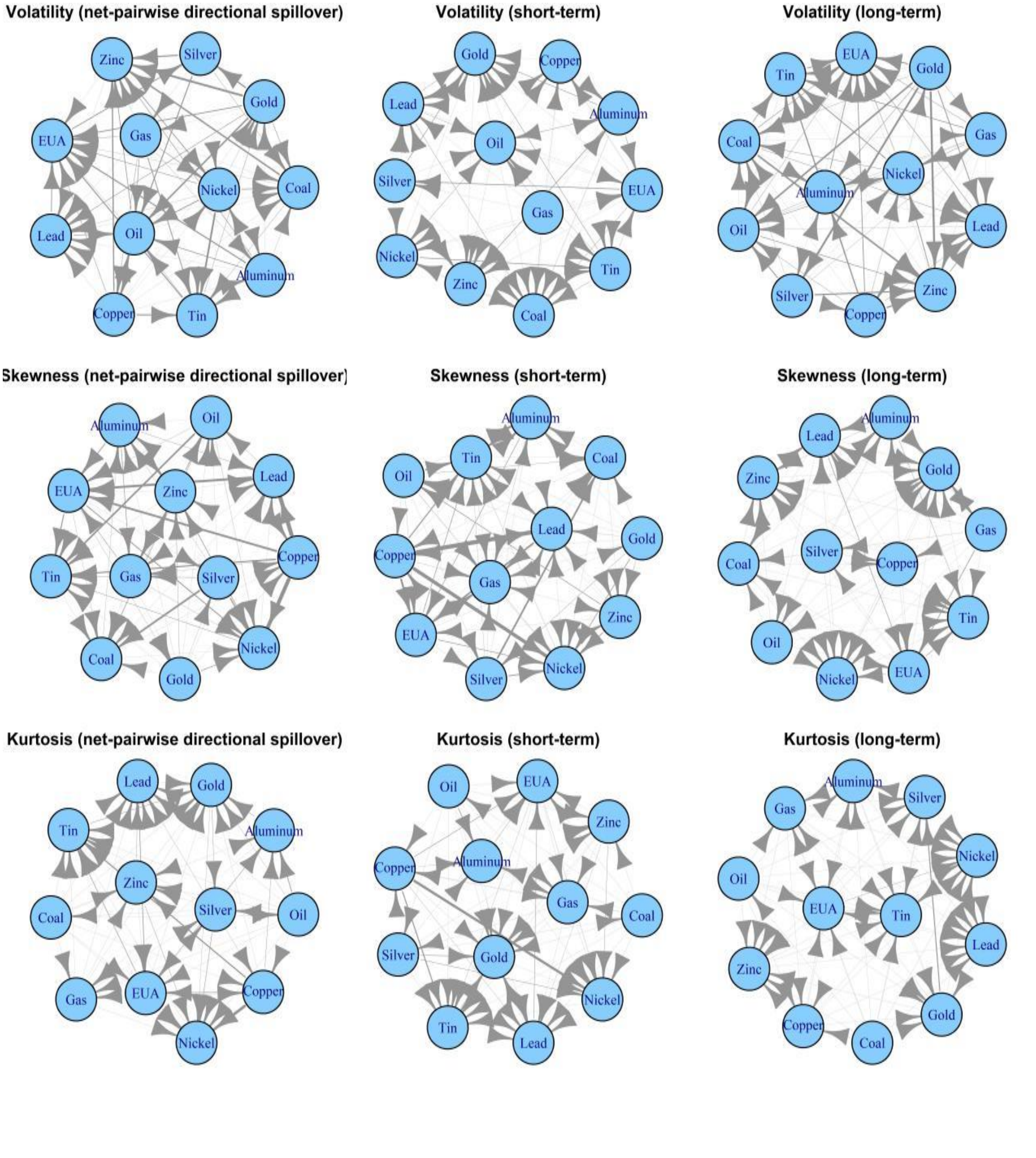

Supplementary Figure 6: Overall volatility spillover index using different rolling-window sizes.

In order to avoid extreme values from masking other period trends, we drew the plots with the monthly mean of dynamic total connectedness. The top panel “Overall spillovers” is the dynamic total spillover index of the DY model. “Short-term” and “Long-term” is the dynamic frequency connectedness on the band:3.14 to 0.14 and 0.14 to 0 of BK model, respectively.

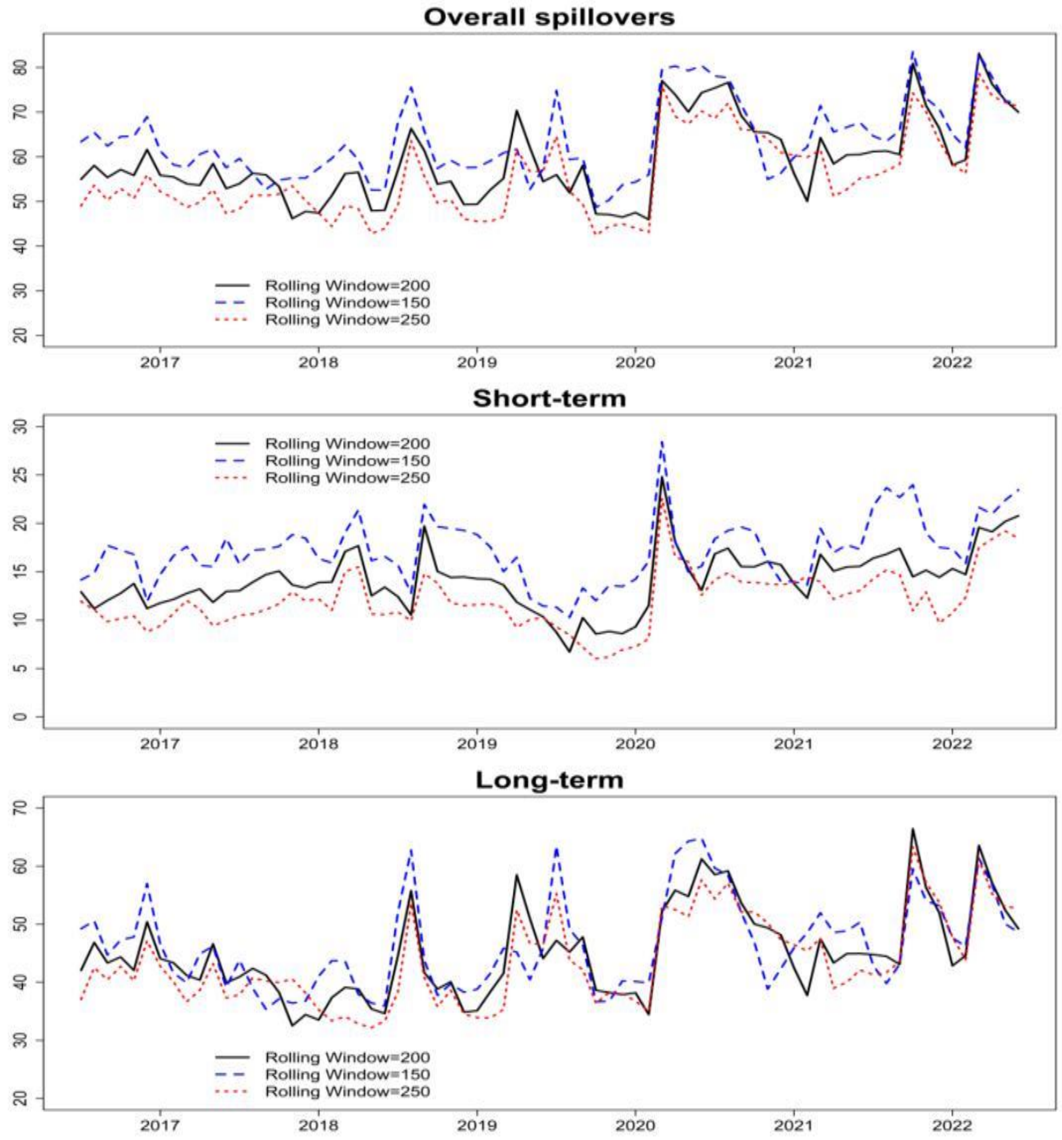

Supplementary Figure 7: Coefficient Comparisons for the quantile-on-quantile (QQ) and

quantile regression (QR) method of the impact of physical risk index(PRI) on total spillover index(TCI). “Overall spillovers” is the dynamic total spillover index of the DY model. The “short-term” and “long-term” is the dynamic frequency connectedness on the band:3.14 to 0.14 and 0.14 to 0 of BK model, respectively. The top panels show the volatility, skewness, and kurtosis spillover effects in different frequencies, such as “Volatility (short-term)” represents the coefficients got by the impact of PRI on TCI in the short-term horizon based on QQ and QR model.

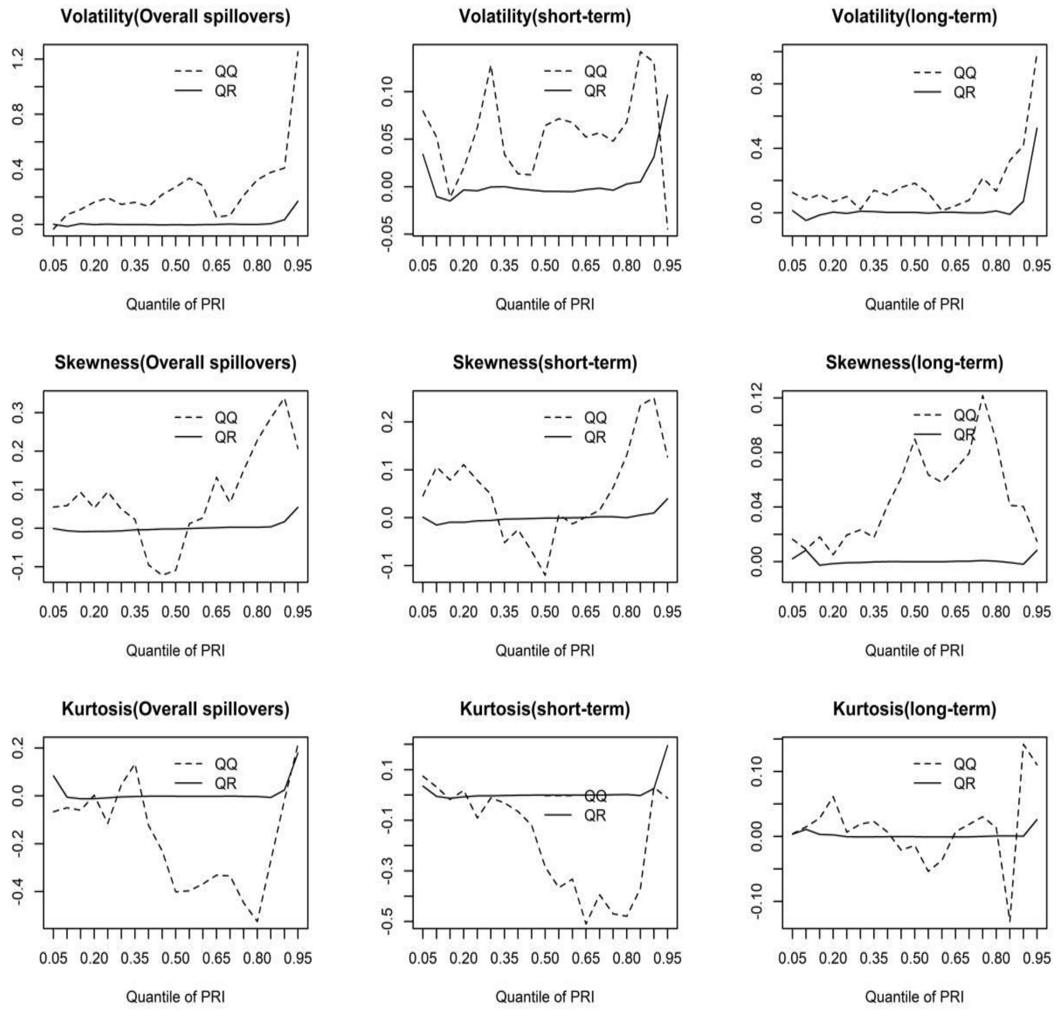

Supplementary Figure 8: Coefficient Comparisons for the quantile-on-quantile (QQ) and quantile regression (QR) method of the impact of transfer risk index(TRI) on total spillover index(TCI). "Overall spillovers" is the dynamic total spillover index of the DY model. The "short-term" and "long-term" is the dynamic frequency connectedness on the band:3.14 to 0.14 and 0.14 to 0 of BK model, respectively. The top panels show the volatility, skewness, and kurtosis spillover effects in different frequencies, such as "Volatility (short-term)" represents the coefficients obtained by the impact of TRI on TCI in the short-term horizon based on QQ and QR model.

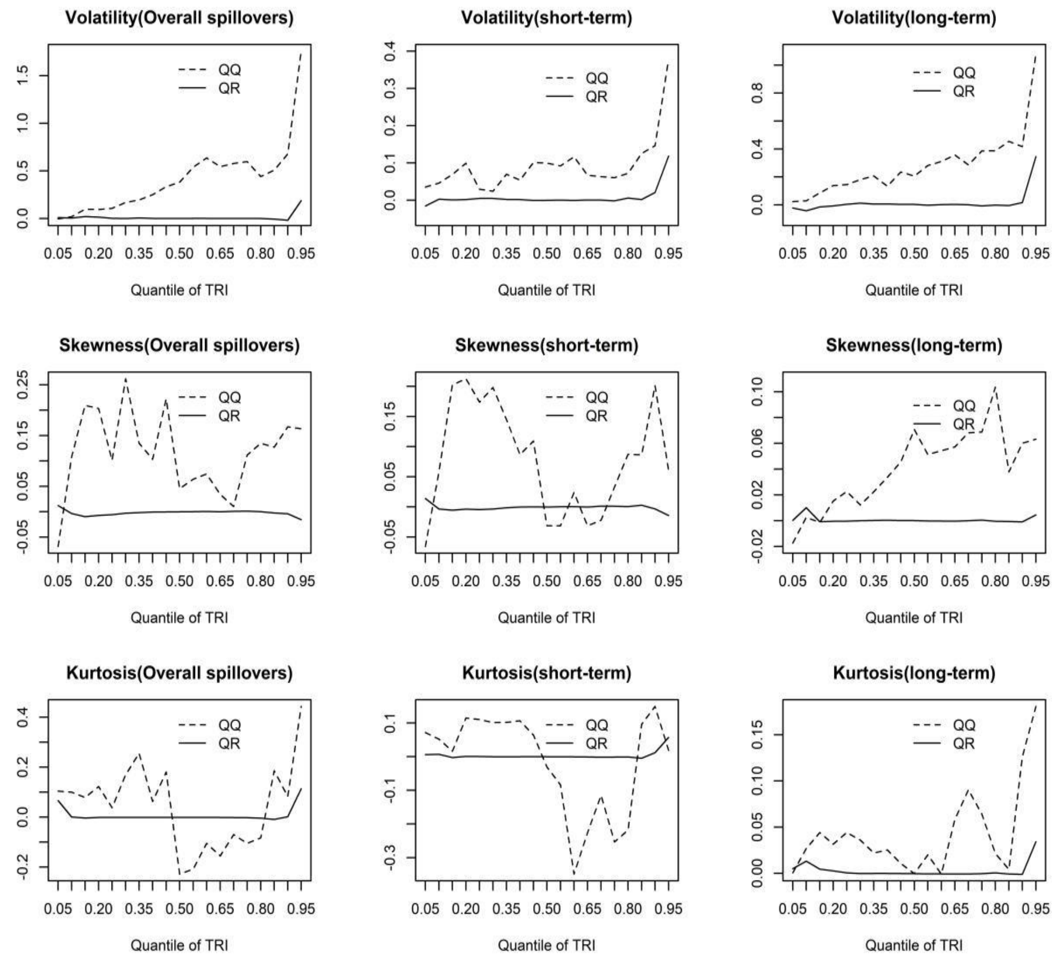

Supplementary Figure 9: Heat map of pairwise correlations of each market's returns. The numbers represent the pairwise correlation between pairs of assets, and the darker color represents a stronger correlation.

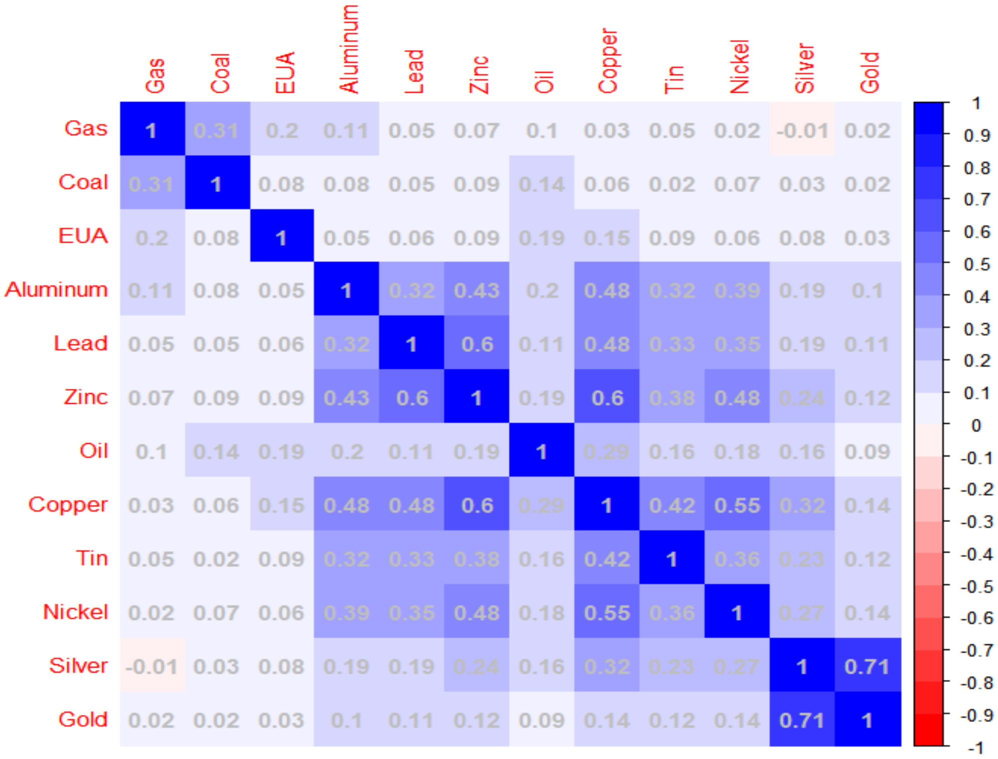

Supplementary Reference

1. Jarque, C. M. & Bera, A. K. Efficient tests for normality, homoscedasticity and serial independence of regression residuals. Econ. Lett. **6**, 255-259 (1980).
2. Dickey, D. & Fuller, W. Distribution of the estimators for autoregressive time series with a unit root. J. Am. Stat. Assoc. **74**, 427-431(1979).
3. Baur, D. G. & Oll, J. Bitcoin investments and climate change: A financial and carbon intensity perspective. Financ. Res. Lett. **47**, 102575 (2022).
4. Chen J., Wang Y. & Ren X. Asymmetric effect of financial stress on China's precious metals market: Evidence from a quantile-on-quantile regression. Res. Int. Bus. Financ. **64**, 101831 (2023).
5. Ozturk, S.S., Demirer, R. & Gupta R. Climate uncertainty and carbon emissions prices: The relative roles of transition and physical climate risks. Econ. Lett. **217**, 110687 (2022).
6. Yan, W-L. & Cheung, A. The dynamic spillover effects of climate policy uncertainty and coal price on carbon price: Evidence from China. Financ. Res. Lett. **53**, 103400 (2023).
7. Xie Q., Hao J. & Zheng X. Carbon price prediction considering climate change: A text-based framework. Econ. Anal. Policy **74**, 382-401(2022).
8. Guo, J., Long, S. & Luo, W. Nonlinear effects of climate policy uncertainty and financial speculation on the global prices of oil and gas. Int. Rev. Financ. Anal. **83**, 102286 (2022).
9. Liang, C., Umar, M., Ma, F. & Huynh, T. L. Climate policy uncertainty and world renewable energy index volatility forecasting. Technol. Forecast. Soc. **182**, 121810 (2022).
10. Nam, K. Investigating the effect of climate uncertainty on global commodity markets. Energ. Econo. **96**,

105123 (2021).

11. Gupta, R. & Pierdzioch, C. Climate risks and forecastability of the realized volatility of gold and other metal prices. Resour. Policy **77**, 102681 (2022).
12. Karmakar, S., Gupta, R., Cepni, O. & Rognone, L. Climate risks and predictability of the trading volume of gold: evidence from an INGARCH model. Resour. Policy **82**, 103438 (2023).
13. Salisu, A. A., Olaniran, A. & Lasisi, L. Climate risk and gold. Resour. Policy 82, 103494 (2023).
14. Babiker, M. H. Climate change policy, market structure,and carbon leakage. J. Int. Econ. **65**,421-445(2005).
15. Watari, T., Nansai, K. & Nakajima, K. Contraction and convergence of in-use metal stocks to meet climate goals. Global Environ. Chang. **69**, 102284 (2021).
16. Tang, C., Sprecher, B., Tukker, A. & Mogollón, J. M. The impact of climate policy implementation on lithium, cobalt and nickel demand: The case of the Dutch automotive sector up to 2040. Resour. Policy **74**, 102351(2021).
17. Shang, Y., Han, D., Gozgor, G., Mahalik, M. K. & Sahoo, B. K.The impact of climate policy uncertainty on renewable and non-renewable energy demand in the United States. Renew. Energ. **197**, 654-667 (2022).
18. Lam, C.K.C. et al. Impact of climate change and socioeconomic factors on domestic energy consumption: The case of Hong Kong and Singapore. Energy Rep. **8**, 12886-12904 (2022).
19. Khalfaoui, R. et al. How do climate risk and clean energy spillovers, and uncertainty affect US stock markets?. Technol. Forecast. Soc. **185**, 122083 (2022).
20. Mao, X., Wei, P. & Ren, X. Climate risk and financial systems: A nonlinear network connectedness analysis. J. Environ. Manage. 340, 117878 (2023).
21. Yuan, N. & Yang, L. Asymmetric risk spillover between financial market uncertainty and the carbon market: A GAS-DCS-copula approach. J. Clean. Prod. **259**, 120750 (2020).
22. Xu, L., Wu, C., Qin, Q. & Lin, X. Spillover effects and nonlinear correlations between carbon emissions and stock markets: An empirical analysis of China's carbon-intensive industries, Energ. Econ. **111**, 106071 (2022).
23. Zhang, J., Hassan, K., Wu, Z. & Gasbarro, D. Does corporate social responsibility affect risk spillovers between the carbon emissions trading market and the stock market. J. Clean. Prod. **362**, 132330 (2022).
24. Reboredo, J.C. Volatility spillovers between the oil market and the European union carbon emission market. Econ. Model. **36**, 229-234 (2014).
25. Balcılar, M., Demirer, R., Hammoudeh, S. & Nguyen, D.K. Risk spillovers across the energy and carbon markets and hedging strategies for carbon risk. Energ. Econ. **54**, 159-172 (2016).
26. Zhang, Y.J. & Sun, Y.F. The dynamic volatility spillover between european carbon trading market and fossil energy market. J. Clean. Prod. **112**, 2654-2663(2016).
27. Ji, Q., Zhang, D. & Geng J. Information linkage, dynamic spillovers in prices and volatility between the carbon and energy markets. J. Clean. Prod. **198**, 972-978 (2018).
28. Xu, Y. Risk spillover from energy market uncertainties to the Chinese carbon market. Pac-Basin. Financ. J. **67**,101561(2021).
29. Adekoya, O. B., Oliyide, J. A. & Noman, A. The volatility connectedness of the EU carbon market with commodity and financial markets in time- and frequency-domain: The role of the U.S. economic policy uncertainty. Resour. Policy **74**, 102252(2021).
30. Tian, T., Lai, K-H. & Wong C.W.Y. Connectedness mechanisms in the "Carbon-Commodity-Finance" system: Investment and management policy implications for emerging economies. Energ. Policy **169**, 113195(2022).
31. Tiwari, A. K., Abakah, E. J. A., Gabauer, D. & Dwumfour, R. A. Dynamic spillover effects among green bond, renewable energy stocks and carbon markets during COVID-19 pandemic: implications for hedging and investments strategies. Glob. Financ. J. **51**, 100692 (2022).

32. Diebold, F.X. & Yilmaz, K. Better to give than to receive: predictive directional measurement of volatility spillovers. Int. J. Forecasting **28**, 57-66 (2012).
33. Diebold, F.X. & Yilmaz, K. On the Network Topology of Variance Decompositions: Measuring the Connectedness of Financial Firms. J. Econometrics **182,** 119-134 (2014).
34. Baruník, J. & Kehlík, T. Measuring the frequency dynamics of financial connectedness and systemic risk. J. Financ. Economet. **16**, 271-296 (2018).
35. Ando, T., Greenwood-Nimmo, M. & Shin, Y. Quantile Connectedness: Modeling Tail Behavior in the Topology of Financial Networks. Manage. Sci. **68**, 2401-2431(2022).
36. Wang, X. X. & Wang, Y. D. Volatility Spillovers between Crude Oil and Chinese Sectoral Equity Markets: Evidence from a Frequency Dynamics Perspective. Energ. Econ. **80**, 995-1009 (2019).
37. Jiang, W. & Chen, Y. The time-frequency connectedness among metal, energy and carbon markets pre and during COVID-19 outbreak. Resour. Policy **77**,102763(2022).
38. Chen, J., Liang, Z., Ding, Q. & Liu, Z. Quantile connectedness between energy, metal, and carbon markets. Int. Rev. Financ. Anal. **83**, 102282 (2022).
39. Yang, C., Lei X. & Shi, B. Spillovers among China's precious and industrial metals markets: Evidence from higher moments and jumps. T. Nonferr. Metal. Soc. **32**, 1362-1384 (2022).
40. Cui, J. & Maghyereh, A. Higher-order moment risk connectedness and optimal investment strategies between international oil and commodity futures markets: Insights from the COVID-19 pandemic and Russia-Ukraine conflict. Int. Rev. Financ. Anal. **86**, 102520 (2023).
41. Zhou, Y., Wu, S. & Zhang, Z. Multidimensional risk spillovers among carbon, energy and nonferrous metals markets: Evidence from the quantile VAR network. Energ. Econ. **114**, 106319(2022).
42. Bouri, E., Lei, X., Xu, Y. & Zhang, H. Connectedness in implied higher-order moments of precious metals and energy markets. Energy **263**, 125588 (2023).
43. Jiang, W. & Chen, Y. The time-frequency connectedness among carbon, traditional/new energy and material markets of China in pre-and post-COVID-19 outbreak periods. Energy **246**, 123320 (2022).
44. Qi, H., Wu, T., Chen, H. & Lu, X. Time-frequency connectedness and cross-quantile dependence between carbon emission trading and commodity markets: Evidence from China. Resour. Policy **82**, 103418 (2023).
